# An automated calibration method for non-see-through head mounted displays

**DOI:** 10.1016/j.jneumeth.2011.05.011

**Published:** 2011-08-15

**Authors:** Stuart J. Gilson, Andrew W. Fitzgibbon, Andrew Glennerster

**Affiliations:** a Department of Physiology, Anatomy and Genetics, University of Oxford, Oxford, UK; b Microsoft Research Ltd., Cambridge, UK; c School of Psychology and Clinical Language Sciences, University of Reading, Reading, UK

**Keywords:** Head mounted display, Non-see-through, Calibration, Photogrammetry, Immersive virtual reality

## Abstract

Accurate calibration of a head mounted display (HMD) is essential both for research on the visual system and for realistic interaction with virtual objects. Yet, existing calibration methods are time consuming and depend on human judgements, making them error prone, and are often limited to optical see-through HMDs. Building on our existing approach to HMD calibration Gilson et al. (2008), we show here how it is possible to calibrate a non-see-through HMD. A camera is placed inside a HMD displaying an image of a regular grid, which is captured by the camera. The HMD is then removed and the camera, which remains fixed in position, is used to capture images of a tracked calibration object in multiple positions. The centroids of the markers on the calibration object are recovered and their locations re-expressed in relation to the HMD grid. This allows established camera calibration techniques to be used to recover estimates of the HMD display's intrinsic parameters (width, height, focal length) and extrinsic parameters (optic centre and orientation of the principal ray). We calibrated a HMD in this manner and report the magnitude of the errors between real image features and reprojected features. Our calibration method produces low reprojection errors without the need for error-prone human judgements.

## Highlights

► Correctly calibrating a head-mounted display (HMD) is critical for the kinds of virtual reality applications used by visual neuroscientists. ► We propose a method that produces these calibrations accurately and quickly. ► Unlike methods proposed by others, ours requires no error-prone human judgements. ► Our method permits the collection of large numbers of samples for the calibration data. We show how number of samples affects calibration accuracy. ► Can be used for the more popular non-see-through HMDs, which, traditionally, have been neglected. Existing calibration techniques work only with optical-see-through HMDs.

## 1 Introduction

A head mounted display (HMD) can be modelled in a similar way to a conventional camera. Like a camera, it has both *intrinsic* parameters – focal length, aspect ratio, centre pixel – and *extrinsic* parameters – position of the optic centre and orientation of the principal ray. It also has an *image plane*, upon which pixels are drawn which represent the rays of light from the scene striking the virtual film. Collectively, these define a set of *projection parameters* which determines how the vertices of virtual objects are projected onto the image plane.

The issue, then, is to find the projection parameters for each eye's display in a HMD. HMD manufacturer specifications tend to be inadequate for this task, so the only other solution is to attempt to measure these display properties. Unlike calibrating a monitor display, it is usually difficult to get sufficient physical access to a HMD display in order to make accurate measurements. Instead, we describe here a method based on photogrammetry (camera calibration) techniques.

HMDs fall into two categories: see-through and non-see-through. Of the see-through variety, there are two sub-categories: optical-see-through and video-see-through. Video-see-through displays are very popular in augmented reality applications, where a video camera mounted within the HMD sends digitized images of the real world to the graphics computer, which can then overlay computer graphics onto the images before sending them to the HMD to be displayed to the observer. Such displays are generally straight-forward to calibrate (Tuceryan et al., 1995; Azuma et al., 2001), since the issue of calibrating a conventional camera is well understood (Hartley and Zisserman, 2001). However, the optic centre of the camera is not at the observer's eye, and the resulting calibrated display will differ from that which the observer would see if they removed the HMD. For some applications, this discrepancy is acceptable (e.g., navigation, gaming, architectural walk-throughs), while for other applications involving interaction with real and virtual objects the offset between hand and eye may be detrimental to the task.

Optical-see-through displays generally use a half-silvered mirror placed in front of the observer's eyes, with a display device (cathode-ray tube or liquid crystal) mounted on the HMD. The half-silvered mirror permits rays of light from the real world to reach the observer, while also reflecting images from the display device. The observer sees a composite of the two sources, but with several limitations. Notably, the computer graphics (CG) image is effectively blended with the real world image and, as such, can never completely obliterate the real world. Hence, making virtual objects occlude real ones is impossible. Also, dark details in the CG image will become washed out by bright areas of the real world. Of more relevance here, is that there is no digital record of the rays entering the HMD optics and, so, existing camera calibration methods cannot be used. Critically, without an accurate calibration, virtual objects will not register precisely with real objects, making optical-see-through a poor choice for augmented reality.

Non-see-through HMDs usually place the display device directly in front of the observer's eye, and are thus optically much simpler than either of the other two types of HMD. This does not make them any easier to calibrate, though. While the real world is not visible to the observer, and so registering virtual objects with real world ones is not an issue, a correct calibration is still important. Failure to calibrate correctly can lead to observers misinterpreting the virtual world (for example, they often underestimate distances to objects which can be a symptom of an incorrect calibration (Kuhl et al., 2009)). Inadequate calibration can also lead to users experiencing premature fatigue and possible onset of nausea (Mon-Williams et al., 1993,1998; Regan, 1995).

Thus, there is a demand for a reliable calibration procedure for both optical-see-through and non-see-through HMDs. Several methods of calibration that have been described previously have relied on human judgements. For non-see-through HMDs this involves either the observer removing the headset and comparing the widths or locations of objects presented in the real world with those shown in the headset (Kuhl et al., 2009) or judging the separation of features in the HMD image with an after-image produced by a bright flash (Rinalducci et al., 1996). Both types of method suffer from the inevitable imprecision and inaccuracy of human judgements. These methods were also designed to calibrate only a restricted range of parameters (e.g., horizontal and vertical scale, pitch and pin-cushion distortion).

Optical see-through HMDs have the advantage that the real world and computer-generated image can be viewed simultaneously. In the literature on optical see-through HMDs, the most extensively covered calibration method, SPAAM (single point active alignment method), uses a human observer to calibrate the display by wearing the HMD and positioning their head in order to align HMD image points with real world objects whose locations are known (Tuceryan et al., 2002). When this alignment is achieved, the HMD position and pose is recorded from the tracker, and the procedure is repeated with more image/world coordinate pairs until sufficient data has been gathered to estimate the projection parameters. This is a time-consuming process, requiring a skilled observer to make numerous, potentially erroneous judgements. Also, there can be high variability in the results due to the difficulty of performing such an alignment task with a free-moving head. Finally, images in the HMD are at a fixed accommodative distance but it is desirable to match image objects with real world objects at a range of distances in order to estimate the projection parameters correctly. Human observers can find it difficult to match the visual direction of real and virtual objects at substantially different distances (and, hence, with different accommodative demands).

(Owen et al., 2004) used a calibration method that at first sight appears similar to ours, with at least one fundamental difference. In our method, all real-world coordinates (of both HMD location and visible markers) are reported by a single tracking system, which obviates the need for error-prone human measurements such as are used by Owen et al. (2004). Unlike Owen et al. (2004) and the majority of other HMD calibration papers, we provide a quantitative evaluation (i.e., root-mean-square error) of the extent to which our calibration has been successful.

We have described previously a calibration method applicable to see-through HMDs (Gilson et al., 2008). Here, we describe a method suitable to *non-see-through* HMDs, thus making our method appropriate for the majority of HMDs currently used for virtual reality applications. In the current method, we used a dynamically tracked object which the user could move freely within the volume visible through the HMD display. This made it easier for the user to cover a wide range of the HMD field of view and hence to obtain a more complete and accurate calibration than would be obtainable with a small number of statically positioned objects.

## 2 Methods

**Fig. 1:**
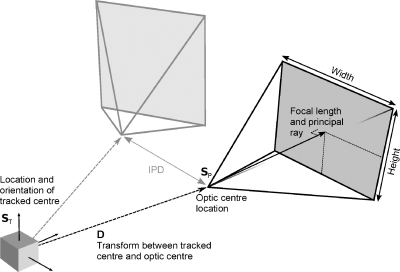
The purpose of calibration is to find values for the displays’ intrinsic parameters, and also the position and orientation of the displays with respect to the HMD's tracked centre.

Our aim was to find estimates of the intrinsic and extrinsic matrices which define the HMD display (shown pictorially in Fig. 1). The extrinsic matrix describes the location of the optic centre of the HMD display and the orientation of the principal ray (in world coordinates):

(1) $$\text{S}=\left[\begin{array}{cc} \text{R} & {\text{T}}^{T}\\ 0 & 1 \end{array}\right]$$

where **R** is a 3 × 3 rotation matrix and **T** is a 1 × 3 translation matrix, i.e., 6 extrinsic parameters.

The intrinsic matrix (Hartley and Zisserman, 2001) comprises the focal length (*f*, in both horizontal and vertical directions, thus denoting aspect ratio), centre pixel location (*c*), and image skew (*s*):

(2) $$\text{K}=\left[\begin{array}{ccc} f_{x} & s & c_{x}\\ 0 & f_{y} & c_{y}\\ 0 & 0 & 1 \end{array}\right]$$

These 11 parameters define a linear projection model, transforming 3D coordinates of virtual objects into image space. In order to obtain projected pixel coordinates (necessary for rendering), we reformulate **K** into a format used in computer graphics languages (like OpenGL):

(3) $$\text{P}=\left[\begin{array}{cccc} \frac{2\times \text{ncp}}{\text{right}-\text{left}} & s & \frac{\text{right}+\text{left}}{\text{right}-\text{left}} & 0\\ 0 & \frac{2\times \text{ncp}}{\text{top}-\text{bottom}} & \frac{\text{top}+\text{bottom}}{\text{top}-\text{bottom}} & 0\\ 0 & 0 & -\frac{\text{fcp}+\text{ncp}}{\text{fcp}-\text{ncp}} & -\frac{2\times \text{fcp}\times \text{ncp}}{\text{fcp}-\text{ncp}}\\ 0 & 0 & -1 & 0 \end{array}\right]$$

where:

$$\begin{array}{c} \text{left}=-\text{ncp}\times \frac{c_{x}}{f_{x}},\;\text{right}=\text{ncp}\times \frac{w-c_{x}}{f_{x}},\\ \text{bottom}=-\text{ncp}\times \frac{c_{y}}{f_{y}},\;\text{top}=\text{ncp}\times \frac{h-c_{y}}{f_{y}} \end{array}$$

define the borders of the frustum's near clipping plane (*w* and *h* are the pixel width and the height of the graphics viewport, and ncp and fcp are the near- and far-clipping planes – these are application specific and not covered further here).

Briefly, the calibration procedure was as below:

1. The HMD was rigidly mounted on a stable table, and it's position and orientation were recorded by the tracking system.
2. The camera was placed inside the HMD such that the camera was approximately fronto-parallel to the HMD display, and could capture as much of one of the displays as possible.
3. A chequerboard grid image was displayed in the HMD display, and captured by the camera. The image was post-processed to locate and record the grid vertices in the camera image.
4. The HMD was carefully moved away from the camera, without moving the camera or table.
5. The calibration object was then waved around within the field of view of the camera. The centroids of the markers on the calibration object were extracted from the camera images in real time. The 3D location of the same markers were also recorded by the tracking system.
6. The 3D marker positions and the 2D image centroids formed the input to the off-line calibration calculations.
7. Typically, this procedure would then be repeated for the other HMD display, although we only describe calibration of one display here.

To capture images for calibration, we used an AVT Pike (1280 × 1024 pixels resolution, ≈65° field of view). The camera was configured to have the smallest lens aperture possible to maximize its depth of field, while still enabling sufficient illumination for the necessary image processing. The shutter time was also minimized as much as illumination allowed, to increase the frame rate of the images and to minimize temporal blurring between frames of any moving objects.

**Fig. 2:**
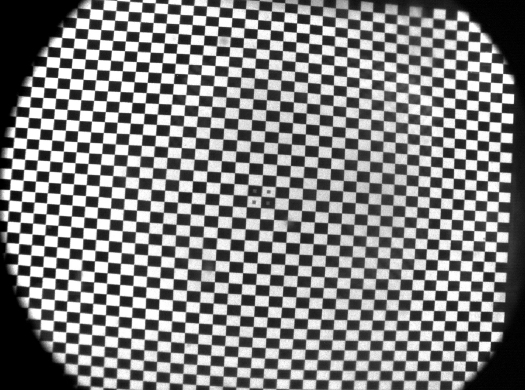
Chequerboard pattern displayed in the HMD and captured by the camera. Note spots indicating the logical centre of HMD display. The vignetting of the grid seen here was due to the camera lens.

We calibrated an nVis SX111 HMD, which has a nominal 76° horizontal field of view in each display, giving a binocular field of view of ≈102° with 66% stereo overlap. Each display comprises 1280 × 1024 pixels and refreshes at 60 Hz. The camera was positioned inside the independently supported HMD in such a way that it could capture as much of the HMD image as possible. The location and pose of the HMD, **S**_T_, was recorded using a 6 degree-of-freedom real time optical tracking system (Vicon Motion Systems MX3). The HMD displayed a simple chequerboard pattern (41 × 41 vertices) and an image of this was captured using the camera (Fig. 2). The vertices of the chequerboard in this image were extracted using image processing. Using a salient feature in the middle of the HMD image, we were able to relate the known vertices of the HMD chequerboard to corresponding vertices in the camera image.

This allowed us to generate a mapping between camera image and HMD coordinates. If the HMD vertices are denoted by:

(4) $${\text{g}}^{\text{HMD}}=\{\left(x_{i}^{\text{HMD}},y_{i}^{\text{HMD}}\right)|i=1...1681\}$$

where $x_{i}^{\text{HMD}}$ and $y_{i}^{\text{HMD}}$ are HMD coordinates, then, for each vertex *i* there exists:

(5) $${\text{g}}^{\text{CAM}}=\{\left(x_{i}^{\text{CAM}},y_{i}^{\text{CAM}}\right)|i=1...1681\}$$

where $x_{i}^{\text{CAM}}$ and $y_{i}^{\text{CAM}}$ are the coordinates of the corresponding vertex in camera coordinates. This allowed any camera coordinate to be converted to a HMD coordinate using interpolation. If ${\text{x}}_{t}^{\text{CAM}}$ denotes a real world point captured by the unmoved camera at time *t*, then we found the smallest triangle of the chequerboard that encompassed it, whose vertices we denote **g**_*i*_, **g**_*h*_ and **g**_*v*_. We then used linear interpolation to re-express the camera coordinate in HMD coordinates using the basis vectors $\left({\text{g}}_{h}^{\text{CAM}}-{\text{g}}_{i}^{\text{CAM}}\right)$ and $\left({\text{g}}_{v}^{\text{CAM}}-{\text{g}}_{i}^{\text{CAM}}\right)$ and their equivalents $\left({\text{g}}_{h}^{\text{HMD}}-{\text{g}}_{i}^{\text{HMD}}\right)$ and $\left({\text{g}}_{v}^{\text{HMD}}-{\text{g}}_{i}^{\text{HMD}}\right)$ respectively. Expressed in terms of these basis vectors, **x**_*t*_^CAM^ and **x**_*t*_^HMD^ are equivalent points (Gilson et al., 2008).

This step essentially rectifies the camera data and means calibration of the camera itself is not required. The HMD calibration procedure relies only on the assumption that the camera coordinates can be mapped onto HMD coordinates by a linear (affine) mapping within the region of a single square of the the chequerboard.

**Fig. 3:**
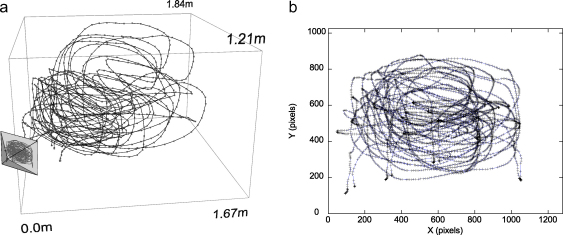
Plots of the 3D marker location and corresponding 2D centroid locations extracted from the camera images. (a) Plot of 3D marker locations (circles, every fifth location shown for clarity) as the calibration object was moved along a trajectory (grey line). Camera only shown schematically, since at this stage the viewing parameters are not known. (b) 2D marker centroids extracted from camera images as the object, consisting of 4 markers, was moved. The data points are shown linked to illustrate the trajectory the markers were moved through, but the ordering information is not needed for calibration since each data point is entirely independent.

We removed the HMD from the camera and – crucially – ensured that the camera did not move. The camera then captured frames of the real world in which we moved a calibration object along a random trajectory within the field of view of the camera. The operator's objective when generating the trajectory was to ‘paint’ as much as possible of the camera image with projections of the markers. In addition, it is beneficial for the accuracy of the calibration results to include as wide a depth range as possible in the trajectory (Fig. 3). The calibration object consisted of several rigidly positioned markers forming an asymmetric planar pattern. The asymmetry allowed the Vicon tracking system to report the object's position unambiguously. By using a number of markers on a rigid object, the Vicon tracking system could report each marker's location with greater accuracy than if just one marker was used. Our calibration software extracted the 2D centroids of the markers from the camera images in real time, while also recording the markers’ 3D locations reported by the Vicon tracker.

Aside from being attached to the same physical object, the data from each marker is treated as entirely independent of the others in the subsequent stages of calibration, so we concatenated their coordinates together to form one large trajectory. For simplicity, the following explanation refers to this compound trajectory as if it were generated by just one marker.

We thus obtained, for each trajectory, approximately 8000 quintuples $\left[{\text{x}}_{t}^{\text{CAM}}{\text{X}}_{t}\right]$ representing the instantaneous location of the marker at any moment (**X**_*t*_) and its projection on the camera image $\left({\text{x}}_{t}^{\text{CAM}}\right)$. Before these coordinates could form the input to the camera calibration routine, all 2D image locations were transformed into HMD coordinates using the basis vectors described above, to give ${\text{x}}_{t}^{\text{HMD}}$. This was a critical step, since without this, the subsequent photogrammetry would produce an intrinsic model of the camera, not the HMD.

We computed initial values for the intrinsic and extrinsic matrices by finding a single homography that mapped ${\text{x}}_{t}^{\text{HMD}}$ onto the corresponding **X**_*t*_ [Hartley and Zisserman, 2001, page 92]. The resulting estimates for focal length, aspect ratio, centre pixel, optic centre location and principal ray direction were then used as a starting point for a simplex minimisation (Lagarias et al., 1998). The cost function was the reprojection error – that is, the root-mean-square (RMS) difference (in pixels) between the original projections **x**^HMD^ and the new projections computed by:

(6) $$(x_{t},y_{t},z_{t},w_{t})=\text{P}{\text{S}}_{\text{P}}{\left[{\text{X}}_{t}1\right]}^{\text{T}}$$

(where the depth component, *z*_*t*_, of the homogeneous coordinate can be discarded leaving a simple difference vector between ${\text{x}}_{t}^{\text{HMD}}$ and (*x*_*t*_, *y*_*t*_)). The matrix **S**_P_ is the location and pose of the camera, estimated by calibration, in absolute tracker coordinates. However, we needed to know these parameters in coordinates relative to the tracked HMD position, so that the correct image could be rendered for any HMD position or orientation. We thus computed **D** as the single transform between HMD tracked centre and the HMD display:

(7) $$\text{D}={\text{S}}_{\text{P}}{\text{S}}_{\text{T}}^{-1}$$

This simplicity arises from the fact that **S**_P_ and **S**_T_ are in the same coordinate frame. We now have a projection matrix (of the HMD display) and a modelling matrix which can be used directly in a 3D programming language such as OpenGL by post-multiplying it with the modelling matrix from the tracker:

// Switch to intrinsic (projection) matrix mode.
glMatrixMode(GL_PROJECTION);
// Load intrinsic matrix, P.
glLoadMatrix(P);
// Switch to extrinsic (modelling) matrix.
glMatrixMode(GL_MODELVIEW);
// Load HMD_to_optic_centre transform.
glLoadMatrix(D);
// Incorporate tracker transform.
glMultMatrix(S_T);

This procedure was repeated for both displays in the binocular HMD, with each display calibrated independently. In our experience, using this method we have found no need to perform an explicit stereo calibration.

Calibration accuracy was quantified as the root-mean-square reprojection error (in pixels) measured for all marker positions in the trajectory. It may seem counter-intuitive to use reprojections as an error measure here, since a non-see-through HMD has no real-world image in which to make such reprojections. The important point, of course, is that the camera did not move between capturing the HMD chequerboard and the corresponding marker trajectories and, thus, each camera pixel corresponded to the same ray irrespective of whether the HMD was present or not.

## 3 Results

We collected 4 trajectories using the method described above, each consisting of at least 8000 samples. We then physically moved the HMD to a new location within the tracked volume and collected another 4 trajectories. We repeated this procedure until we had acquired 6 sets of 4 trajectories for one HMD display. The importance of moving the HMD to new locations is two-fold. First, the spatial relationship between the HMD and the camera will change each time – the effects of this change will be discussed below. Second, the relocation demonstrates that the calibration method works irrespective of HMD position, orientation, angle of inclination, etc.

**Fig. 4:**
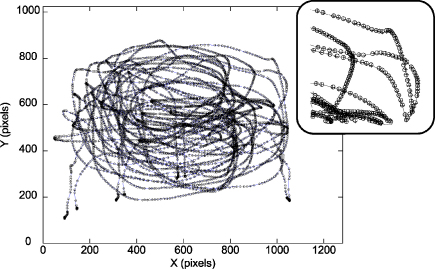
Plot of calibrated solution (circles) for the trajectory shown in Fig. 3 (plus-signs). The inset shows a magnified section, revealing the accurate alignment of the reprojected data. The reprojection error in this case is 0.89 pixels.

We first present a typical calibrated solution from one trajectory. Fig. 4 shows the solution for the trajectory shown in Fig. 3. Here, the 3D marker data (**X**) is reprojected using the calibrated projection parameters to produce a new set of 2D pixel locations, **y** (shown as circles in Fig. 4). Plotting **y** together with **x** (the original marker centroids captured by the camera, shown as plus-signs) reveals the close coincidence between the two. Incorrect estimates of the camera parameters would result in a spatial offset between **y** and **x**. No such systematic offset is evident in Fig. 4. The trajectory was gathered over a wide range of distances and yet the errors are low across the whole trajectory, demonstrating the applicability of the calibration over a large working volume. The reprojection error for this example is 0.89 pixels. For the nVis SX111 HMD, with a calibrated left display of 75° horizontal field of view, this reprojection error represents ≈3.13 arcmin (where each pixel subtends ≈3.5 arcmin). Note that the calibrated horizontal field of view is close to the manufacturer's specification of 76° but using the latter figure would result in a systematic error, greatest at the edges of the display, of over 2 pixels.

**Fig. 5:**
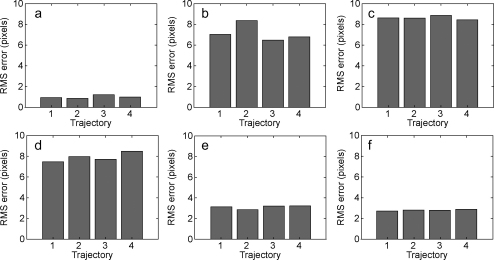
Generalisation of a calibration. (a) Column 1 shows the reprojection error for a calibration generated from ≈8000 samples of marker location. Three other similar trajectories were captured and columns 2–4 show the reprojection error when these trajectories were tested using the calibration computed from the first trajectory. (b)–(f) Show the reprojection error when trajectories recorded from the five other camera locations were tested, again using the calibration computed from the first trajectory.

For any single trajectory, the presence of noise (and possibly other idiosyncratic aspects of the measurement apparatus) will inevitably lead to errors in the estimation of projection parameters. Such incorrect estimation will be evident in higher reprojection errors of *other* trajectories captured under identical conditions (i.e., when the camera is not moved or adjusted). We used the extra 3 trajectories captured for each camera/HMD position to test this by calculating the reprojection errors for the remaining trajectories with that calibration. Fig. 5 shows reprojection errors for the 4 trajectories within each data set when they were tested with the calibration generated from the first trajectory in the first data set (trajectory 1 in Fig. 5a). It is clear that the original calibration generalized well to the other trajectories from the same camera/HMD position, with reprojection errors remaining at about 0.91 ± 0.12 (mean ± standard deviation) pixels for the novel trajectories. This indicates that the calibration was not over-specialized or otherwise influenced by measurement noise in the tracker and image processing coordinates.

We could also measure generalisation across the other 20 trajectories from the other 5 camera positions, which tests quite a distinct aspect of the calibration. Fig. 5b–f illustrates the consequences of testing the original calibration (from Fig. 5a, trajectory 1) but now using trajectories that were collected with the camera in a slightly different location with respect to the HMD display. Each panel shows results for the camera in a different location. It is clear that these trajectories result in a larger reprojection error (up to 9 pixels). The different trajectories *within* each panel (i.e., taken from the same camera location) all have similar reprojection errors but, in each case, they confirm that the calibration is an inappropriate one for these trajectories.

We tested all 24 calibrations using each of the 24 trajectories (576 combinations, not shown). We found that it was always the case that the reprojection errors for the trajectories viewed from the same location as the trajectory used to calibrate the HMD (i.e., cases equivalent to those shown in Fig. 5a) were low compared to those tested with trajectories obtained from a different camera location. Specifically, generalisation within the same camera position had reprojection errors of 0.98 ± 0.01 pixels; for generalisation to other trajectories taken from a different location from the one used for calibration, reprojection errors were on average 6.71 ± 3.05 pixels.

**Fig. 6:**
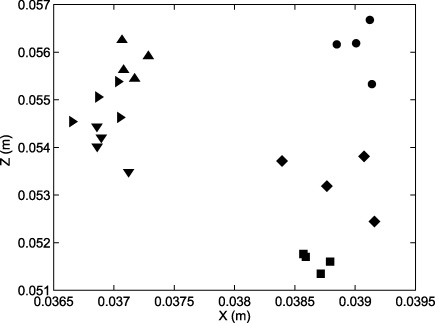
Optic centre locations recovered by calibration from the 24 trajectories plotted relative to the HMD tracked centre (**S**_T_).

Clearly, the effect of physically moving the headset to a different region of the tracked volume necessarily involves the camera being moved in relation to the headset and, critically, altering the spatial relationship between the camera's optic centre and principal ray relative to the HMD display. However, the relationship between the camera and HMD locations is not unbounded – the constraint that the camera must be positioned to capture as much of the HMD grid image as possible means that it will lie within a small region whose centre is close to the HMD display's true optic centre. Further, the camera placement with respect to the HMD display is likely to mimic that of a human user wearing the same HMD: each occasion on which they re-position the HMD will yield a different alignment, but constrained by the requirement to obtain a clear image. The recovered locations of the camera optic centre with respect to the HMD's tracked centre are illustrated in Fig. 6, for all 24 trajectories. Changes in optic centre location of this magnitude would be expected to give rise to errors of the sort shown in Fig. 5b–f. In Section 5, we consider the consequences of such head movements for both see-through and non-see-through HMDs.

**Fig. 7:**
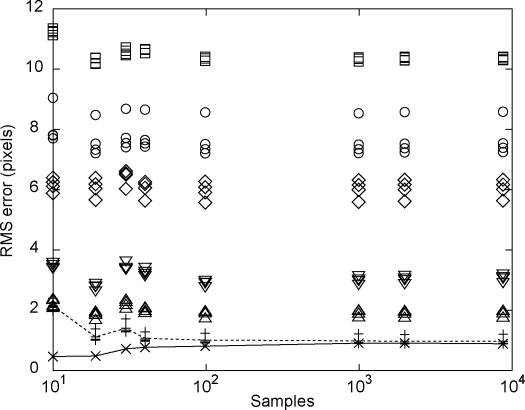
Calibration errors as a function of number of samples used from the tracked object trajectory. The crosses (×) show reprojection errors for a single trajectory. These are plotted against the sample size, with samples picked randomly from the ≈8000 locations of the tracked marker. The plus-signs (+) show how well this calibration generalised to the three different trajectories captured with the same camera position (c.f. Fig. 5a). The open symbols show reprojection errors when this calibration was tested against trajectories taken from the three alternate camera locations (c.f. Fig. 5b–f).

We next considered the number of samples that are required in order to obtain an accurate calibration. Because each sample quintuple of $\left[{\text{x}}_{t}^{\text{CAM}}{\text{X}}_{t}\right]$ is entirely independent of the others, we were able to subsample each of the trajectories obtained to see how calibration accuracy varied with the number of samples used. Fig. 7 shows how reprojection error changed as more samples were made available to the calibration procedure. For each number of samples, a new calibration was generated using that number of samples from one particular trajectory. The reprojection errors for these calibrations are shown by the crosses: these are about 0.5 pixels for the smallest number of samples and rise to about 0.9 pixels for the case when all 8000 samples in the trajectory were used. The other 23 points plotted for a given sample size show how the calibration generalises to other trajectories, i.e. the reprojection errors are shown when other (whole) trajectories are tested using the calibration generated from the (sampled) first trajectory.

For the smallest number of samples tested (10), the algorithm over-fits the data, as is evident from the fact that there is a low reprojection error on the training data but high reprojection errors on the test trajectories. Between 10 and 1000 samples, the reprojection error on the trajectory used to generate the calibration actually deteriorates but this is accompanied by an improvement in the reprojection errors for other trajectories taken from the same camera location (plus signs), as would be expected if the calibration is converging on the correct solution. Above 1000 samples there was no clear improvement in calibration generalisation for other trajectories captured from the same camera location, even when all 4 trajectories were combined (over 32,000 samples, reprojection error of 0.9 pixels). Trajectories taken from other camera locations led to a worse calibration, as we discussed in relation to Fig. 5 but, unlike the trajectories taken from the same camera location, the reprojection errors are relatively unaffected by sample size. Of course, the main point is to determine the number of samples that would be required to achieve a reasonable calibration. From Fig. 7, and confirmed from similar calibrations, it would appear that 1000 samples is reasonable.

**Fig. 8:**
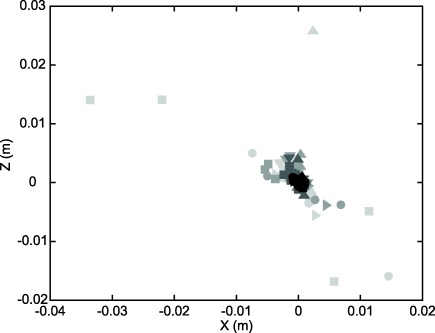
Two components of the HMD optic centre locations, estimated for different numbers of samples for all trajectories from the 6 HMD positions. Light grey symbols represent the results when the calibration is based on only 7 samples; mid grey: 10 samples; dark grey: 20 samples; black: 100 samples. As the number of samples increases, the estimated optic centres cluster more closely around the estimate obtained using the combined trajectories for each HMD position (≈32,000 samples, origin of plot).

Given that the ultimate aim is to recover the 11 projection parameters of the display, it is instructive to plot the change in these as the number of samples increases and reprojection errors drop. Fig. 8 illustrates the **X** and **Z** translation components of the optic centre plotted as a function of the number of samples used in the calibration for the 4 trajectories from the HMD position shown in Fig. 7. It can be seen that, when the sample number is low, the estimates of the optic centre are scattered around the location of the best estimate, obtained with 32,000 samples. This reduction in scatter is accompanied by a relatively modest fall in the reprojection error, from ≈2.0 pixels for 10 samples to ≈1.0 pixels for 32,000 samples. The examples illustrate the advantage of an automatic, camera-based method over those that rely on human judgements of alignment, such as SPAAM (Tuceryan et al., 2002), which are inevitably limited in the number of samples that can be obtained.

Finally, we repeated the estimation of projection parameters including a model of non-linear (radial and tangential) distortions (Heikkilä and Silvén, 1997). The extra 5 parameters were included in the simplex minimisation, and the image corrections were applied to the pixel coordinates obtained in Eq. (6). The fact that data samples were captured at a high density across a wide area of the image meant that non-linear parameters could be well estimated. For the SX111 HMD used here, the non-linear parameters were small and reprojection errors were reduced, on average, by 7%.

## 4 Discussion

In addition to the objective measures of calibration accuracy described above, it is important to consider the implications for humans wearing the HMD. For example, the optic centre of the wearer will never align perfectly with that found by the calibration procedure. As we saw in Fig. 5, movement of the camera relative to the HMD results in larger reprojection errors. Such movement is comparable to that of a human observer wearing a HMD – each usage would change the relative position of their binocular optic centres in relation to the HMD. The issue is complicated by the optics used in HMDs, many of which use an approximation to collimated optics in order to relax the constraint of positioning the observer's eyes directly in line with the display's exit pupils. As a result, some shifts in the position of the eyes’ optic centres relative to the exit pupils still give rise to a clear, focussed image.

The importance of small shifts in the optic centre of the user relative to the HMD display depends on whether the display is see-through or not. For a see-through display, translation of the user's optic centre will result in parallax between features drawn on the HMD screen and real objects viewed through the screen. The extent of this parallax is particularly evident in the large rise in reprojection errors for Fig. 5b–d relative to those in Fig. 5a. We do not know the range of optic centre translations that participants tolerate relative to the HMD but it is likely to be a very much smaller range in augmented reality headsets than in a non-see-through headset because human observers are exquisitely sensitive to relative offsets in the alignment of visual features.

The subjective impression that observers obtain in a non-see-through headset is quite different from an optical see-through headset. If, for example, the simulated inter-ocular separation is changed on a headset while an observer is wearing it, the observer will generally not notice much of a change in the perception of distance, size or stability of objects in the scene despite the fact that, in a see-through display, such changes in simulated inter-ocular separation would cause very noticeable parallax between the real and virtual scenes. It is interesting to speculate about the reasons for this difference. Briefly, it probably implies that observers are not reconstructing the virtual scene when they view a scene with a non-see-through headset (or, indeed, when they view an ordinary scene). The argument is that there is no consistent interpretation of a static scene and fixed camera calibration parameters that could explain that scene so, if they are perceiving a stable scene, they must be doing something other than reconstruction. We have made a similar argument in relation to experiments on an expanding virtual scene (which observers perceive to be stable (Glennerster et al., 2006; Svarverud et al., 2010)). In that case, there *is* a stable interpretation of the images the observer receives but only if they are prepared to accept wildly inaccurate estimates of the optic centre locations, including both inter-ocular separation and translation of the head. More likely, in both the expanding room and the case of manually changing the inter-pupillary distance on a HMD, the reason that the world appears to remain stable is that the visual system is remarkably prone to accepting that this is the case.

A consequence of this difference is that the calibration technique we describe here is probably sufficient, in general, when using a non-see-through HMD, but a more stringent or adaptive method is likely to be required to obtain a highly accurate solution when using an optical-see-through HMD. Broadly, to calibrate more adaptively, two alternatives seem to be available. One possibility is that while the observer is wearing the see-through HMD they must adjust the position of the headset and the optics using a visual alignment procedure until the optic centre of each eye is in the ‘correct’ location, where ‘correct’ means the optic centre for which the HMD is calibrated. This may, in practice, be the simplest solution. An alternative method might be to allow the observer to adjust the HMD until they are comfortable with the view and then try different, precomputed calibrations. They could use an alignment method against a calibration rig to judge which calibration gives the best alignment relative to fixed markers in the scene. This approach has some similarities to the SPAAM procedure described earlier (Tuceryan et al., 2002) and would need to be performed each time the user wears the HMD. It may also be helpful to obtain an independent estimate of the location of the optic centre relative to the headset in order to choose the best pre-computed calibration. Sousa et al. (2010) have described how this can be achieved using a long, tracked, hand-held tube containing cross-hairs at each end which the user repositions until the cross-hairs are aligned. When repeated from several different orientations, these ‘rays’ constrain the estimated location of the optic centre. Nevertheless, it is worth remembering that the human eye does not rotate around it's optic centre, and thus any changes in gaze will change the spatial relationship between the calibration's and observer's optic centres. Until optical-see-through HMDs are able to dynamically adapt to gaze direction, it may not be possible to obtain a truly ‘perfect’ alignment of real and virtual worlds.

## 5 Conclusion

We have presented here an HMD calibration method for non-see-through HMDs, which are the most common type of HMDs used in virtual reality applications. Our method, based on our earlier optical see-through HMDs calibration work (Gilson et al., 2008), provides a quick, reliable and robust method to calibrate each display of an HMD. Unlike existing calibration methods (Tuceryan et al., 2002; Owen et al., 2004), ours does not require error-prone human measurements and provides an objective measure of calibration accuracy.
